# Dysregulation of the DNA Damage Response and *KMT2A* Rearrangement in Fetal Liver Hematopoietic Cells

**DOI:** 10.1371/journal.pone.0144540

**Published:** 2015-12-11

**Authors:** Mai Nanya, Masaki Sato, Kousuke Tanimoto, Minoru Tozuka, Shuki Mizutani, Masatoshi Takagi

**Affiliations:** 1 Department of Pediatrics and Developmental Biology, Tokyo Medical and Dental University, Tokyo, Japan; 2 Analytical Laboratory Chemistry, Graduate School of Health Care Sciences, Tokyo Medical and Dental University, Tokyo, Japan; 3 Department of genomics, Tokyo Medical and Dental University, Tokyo, Japan; Josep Carreras Leukaemia Research Institute, University of Barcelona, SPAIN

## Abstract

Etoposide, a topoisomerase 2 (TOP2) inhibitor, is associated with the development of *KMT2A (MLL)*-rearranged infant leukemia. An epidemiological study suggested that *in utero* exposure to TOP2 inhibitors may be involved in generation of *KMT2A (MLL)* rearrangement. The present study examined the mechanism underlying the development of *KMT2A (MLL)*-rearranged infant leukemia in response to *in utero* exposure to etoposide in a mouse model. Fetal liver hematopoietic stem cells were more susceptible to etoposide than maternal bone marrow mononuclear cells. Etoposide-induced *Kmt2a* breakage was detected in fetal liver hematopoietic stem cells using a newly developed chromatin immunoprecipitation (ChIP) assay. Assessment of etoposide-induced chromosomal translocation by next-generation RNA sequencing (RNA-seq) identified several chimeric fusion messenger RNAs that were generated by etoposide treatment. However, *Kmt2a (Mll)*-rearranged fusion mRNA was detected in *Atm*-knockout mice, which are defective in the DNA damage response, but not in wild-type mice. The present findings suggest that *in utero* exposure to TOP2 inhibitors induces *Kmt2a* rearrangement when the DNA damage response is defective.

## Introduction

The topoisomerase 2 (TOP2) inhibitor etoposide induces DNA double-strand breaks between the S and G2/M phases of the cell cycle. Etoposide is widely used as a chemotherapeutic agent against solid tumors and hematological malignances. However, etoposide induces chemotherapy-associated secondary leukemia, which involves rearrangement of the *KMT2A* (*MLL*) gene on chromosome 11q23 [1]. The KMT2A protein is a transcriptional coactivator that plays an essential role in regulating gene expression during early development and hematopoiesis. Chromosomal translocations involving *KMT2A* are responsible for some cases of *de novo* acute lymphoblastic leukemia (ALL) and acute myeloid leukemia (AML). In addition to their role in chemotherapy-associated secondary leukemia, chromosomal translocations involving *KMT2A* are associated with infant leukemia [2] [3] [4]. In ALL, *KMT2A* translocations are associated with poor clinical outcome [5].

Investigation of identical twin pairs with infant leukemia provided evidence of the *in utero* transfer of leukemic cells from one twin to the other [6], and the *in utero* origin of this cancer was confirmed by retrospective analyses of neonatal blood spots (Guthrie cards) from affected infants [7]. The high concordance rate for leukemia in monozygotic twins and the short latency of the disease suggest that *KMT2A* fusion in fetal hematopoietic stem cells (FL-HSCs) causes infant leukemia. Therefore, determining how *KMT2A* gene alterations occur *in utero* is important. The findings described above suggest the possibility that *KMT2A*-rearranged infant leukemia is caused by transplacental exposure to TOP2 inhibitors. Although it is unusual for a pregnant woman to be directly exposed to drugs such as etoposide, other compounds in the environment may exert similar effects. For example, benzoquinones from cigarette smoke, isoflavones from soybeans, flavonoids from citrus or tea, lignans from flax and sesame seed, some herbal medicines, laxatives such as senna, podophyllin resin, quinolone antibiotics, and some pesticides including certain fungicidals and mosquitocidals can act as TOP2 inhibitors [8]. Indeed, several dietary bioflavonoids induce cleavage of *KMT2A* [9], and epidemiological studies indicate an elevated risk of leukemia in infants exposed *in utero* to DNA-damaging drugs, herbal medicines, dipyrone, and mosquitocidals [8].

To elucidate the etiology of infant leukemia, it would be useful to combine epidemiological and case-based genomic studies with cell-biological analyses. Although several previous studies successfully detected TOP2 inhibitor-dependent *KMT2A* rearrangement *in vitro* [10–12], such rearrangements have not been observed *in vivo*. Furthermore, because the access to human fetuses is limited, no experimental model of *in utero* exposure has been reported to date. To overcome this obstacle, we used a mouse model to investigate how maternal exposure to etoposide affects the *Kmt2a* (*Mll*) gene in fetal hematopoietic cells.

The DNA damage response pathway is critical for the maintenance of genome integrity. For example, spontaneous chromosomal translocation in circulating lymphocytes is observed in ataxia telangiectasia, which is caused by mutation of ataxia telangiectasia mutated (ATM). ATM is a central player in the DNA damage response and exerts its function by phosphorylating a variety of substrates including histone H2AFX (H2AX) [13]. We previously demonstrated that a defective DNA damage response via ATM is required for *KMT2A* rearrangement *in vitro* [14].

In the present study, we showed that *in utero* exposure to a TOP2 inhibitor induces *Kmt2a* breakage in the mouse fetus. In addition, we showed that rearrangements involving the *Kmt2a* gene occur only in mice with defects in the DNA damage response, and not in wild-type animals.

## Materials and Methods

### Mice

This study was performed in strict accordance with the recommendations of the Guide for the Care and Use of Laboratory Animals of the Tokyo Medical and Dental University. C57BL/6 mice were used in the study. Atm-deficient mice (*Atm* ^-/-^) [15] were backcrossed onto the C57BL/6 background for more than 15 generations. Mice were bred in a specific pathogen-free unit in the vivarium of Tokyo Medical and Dental University. Approximately 100 mice were used in this study. Mice were sacrificed using carbon dioxide (CO_2_) according to science council of Japan guidelines on animal experiment. Experimental manipulations and animal care were approved by the Tokyo Medical and Dental University Animal Care and Use Committee (protocol numbers 0140017A and 010018A).

### Etoposide concentration measurement

Fetal liver was homogenized and centrifuged (13,000 rpm for 15 min). Serum fractions were subjected to high-performance liquid chromatography (HPLC) to measure etoposide concentration.

### Fetal liver-derived hematopoietic stem cell (FL-HSC) isolation

Fetal livers were homogenized, and mononuclear cells (MNCs) were isolated using Ficoll-Paque Plus (GE Healthcare, Little Chalfont, UK). Ter119-positive cells were removed from MNCs. Then, CD117^+^ CD45^+^ cells were positively isolated using a MACS system (Miltenyi Biotec, Auburn, CA, USA).

### Flow cytometry

FL-HSCs and maternal bone marrow (mBM) cells were fixed in 1% formaldehyde in PBS for 15 min on ice. After washing in PBS, cells were resuspended in 70% ice-cold ethanol, and then incubated at ˗20°C overnight. The cells were then washed twice with PBS and incubated for 30 min at room temperature (RT) in 1 μl of mouse FcR blocking reagent (Miltenyi Biotec, Auburn, CA, USA) in 50 μl of PBS containing 1% BSA (0.5 μg/50 μl). To detect γH2AX (Serine 139 phosphorylated H2AX), 2 × 10^5^ cells were incubated for 1 h at RT with FITC-conjugated γH2AX antibody (EMD Millipore, Billerica, MA, USA) diluted in PBS/1% BSA. After two washes in PBS, cells were resuspended in 1 ml of PBS containing 5 μg/ml propidium iodide (PI) (Sigma-Aldrich, St Louis, MO, USA) and 200 μg/ml RNase A (Sigma-Aldrich) by stirring for 20 min at 37°C. Flow cytometry was performed on a FACS Calibur instrument (Becton-Dickinson, San Jose, CA, USA). Phospho H3-positive cells were detected using Alexa Fluor 488-conjugated phospho-Histone H3 Serine 10 antibody (EMD Millipore).

### Chromatin immunoprecipitation (ChIP) assay

FL-HSCs were isolated from five pregnant mice on day 13.5 and resuspended in 5 ml of PBS, fixed by addition of 5 ml of 2% formaldehyde in PBS, and incubated for 10 min at RT with rotation. Fixation was quenched by addition of 513 μl of 2.5 M Glycine in PBS (pH 7.0), and the samples were incubated for an additional 5 min at RT with rotation. Cells were washed with PBS twice, and then lysed with cell lysis buffer I (10 mM HEPES [pH 6.5], 10 mM EDTA, 0.5 mM EGTA, 0.25% Triton X-100, and protease inhibitors) for 10 min on ice. After centrifugation and removal of the supernatant, 600 μl of nuclear lysis buffer (50 mM Tris-Cl [pH 8.0], 10 mM EDTA, and 1% SDS) was added, and the samples were incubated for 10 min on ice. The cells were sonicated (four rounds of 10 sec duration, amplitude 6) on ice using BRANSON SONIFIER 250 (Danbury, CT, USA). Samples were centrifuged (13,000 rpm for 15 min), and the supernatant was transferred to a new tube and diluted with five volumes of dilution buffer (1% Triton X-100, 2 mM EDTA, 20 mM Tris-Cl [pH 8.1], and 150 mM NaCl). The diluted supernatant was pre-cleared with 30 μl of protein A-Sepharose coated with salmon sperm DNA and rabbit IgG for 1 h at 4°C with rotation. After pre-clearing, a 1 ml aliquot of supernatant was transferred to a new tube, and protein A Dynabeads (Life Technologies, Carlsbad, CA, USA) coated with rabbit polyclonal γH2AX antibody (EMD Millipore) (1 μg/30 μl Dynabeads) were added; the mixture was then incubated for 2 h at 4°C with rotation. Immunoprecipitated proteins were washed five times with RIPA buffer containing 0.5 M LiCl, followed by two washes with Tris-EDTA (TE) buffer. Antibody/protein/DNA complexes were eluted with 150 μl of elution buffer (0.1 M NaHCO_3_ and 1% SDS) and vortexing; this process was repeated, and both eluates were combined in the same tube. One microliter of RNase A (from 10 mg/ml stock) was added, and the samples were incubated at 37°C for 1 h. To extract DNA, 7.5 μl of proteinase K (from 500 μg/ml stock) was added, and the sample was incubated at 42°C for 3 h. To reverse formaldehyde cross-links, the samples were incubated at 65°C overnight. DNA was purified using the QIAquick PCR Purification Kit (QIAGEN, Hilden, Germany) and eluted in 30 μl of distilled water. PCR primers were as follows: human *BCL9L* region I CTCTGAATCGAGGGATGGAG and GGCCAACCAGATCTCACCTA, human *KMT2A* region II GCAGGCACTTTGAACATCCT and CCAGTTGGTGCTGATTTCCT, region III TGGAAAGGACAAACCAGACC and CACTGCGGGAGATTCAGAGT, region IV CTCTGAATCTCCCGCAGTGT and AGGGCTCACAACAGACTTGG, mouse *Bcl9l* region I CTCTGAATCGAGGGATGGAG and GGCCAACCAGATCTCACCTA, mouse *Kmt2a* region II TTCTCAGGAATTGGAGCCAC and, CGGAATGTGCTAAATGCAGA, region III TGTATGACTATGCACTGGGATTGA and, GAAGGCAATGGGCGGCAG, region IV TGGTTACCTGAATTATGTCCCCAG and GTTCAGGAACTTGCGGCATTTTT. PCR condition was 96°C 30 sec, 60°C 30 sec, 72°C 30 sec, 35 cycle amplification.

### Western blotting

Aliquots of 1 × 10^6^ FL-HSCs isolated from five fetal livers from pregnant mice on day 13.5 were washed with PBS and lysed in RIPA buffer (150 mM NaCl, 1.0% NP-40, 0.1% SDS, 0.1% sodium deoxycholate, 5 mM EDTA, and 10 mM Tris-HCl [pH 7.4]) containing protease inhibitors. Protein concentration was measured using the DC Protein Assay Kit (Bio-Rad, Richmond, CA, USA). After boiling with sample buffer, 30 μg of protein was subjected to SDS-PAGE and transferred to a membrane. Blots were probed with anti-ATM (4D2), anti–phospho-ATM Serine 1981 (Cell Signaling Technology, Danvers, MA, USA), anti-γH2AX (Cell Signaling Technology), or β-actin (Sigma-Aldrich) antibody. Primary antibodies were detected using horseradish peroxidase (HRP)-conjugated anti-mouse secondary antibody (GE Healthcare, Little Chalfont, UK).

### RNA sequencing (RNA-seq)

*Atm* ^+/-^ female mice were crossed with *Atm* ^+/-^ male mice. Pregnant *Atm* ^+/-^ females on day 13.5 were intraperitoneally (IP) injected with saline or 0.5 mg/kg etoposide on 3 consecutive days, and sacrificed 24 h after the final injection. After genotyping, samples were subjected to RNA-seq. Total RNA was extracted from FL-HSCs using Trizol (Life Technologies) or RNeasy (QIAGEN). The integrity and purity of total RNA were assessed by OD260/280 and using an Agilent Bioanalyzer 2100. cDNA (1–2 μg) was generated using the Clontech SmartPCR cDNA kit (Clontech Laboratories, Mountain View, CA, USA) from 100 ng of total RNA, and adaptors were removed by digestion with *Rsa*I. The resultant cDNA was fragmented using a Covaris sonicator (Covaris, Woburn, MA, USA), profiled using an Agilent Bioanalyzer 2100, and subjected to Illumina library preparation using NEBNext reagents (New England Biolabs, Ipswich, MA, USA) or the TruSeq RNA Library Prep Kit (Illumina, San Diego, CA, USA). The quality, quantity, and size distribution of the Illumina libraries were determined using an Agilent Bioanalyzer 2100. The libraries were then subjected to sequencing on an Illumina HiSeq 1500 or HiSeq 2000 according to standard protocols. Paired-end 90 or 100 nucleotide (nt) reads were generated, and the data quality was checked using FASTQC (Babraham Institute, Cambridge, UK).

### Metaphase spread

FL-HSCs from pregnant mice on day 13.5 were enriched from mouse fetal livers using MACS beads (Miltenyi, Bergisch Gladbach, Germany) and cultured with Iscove’s modified Dulbecco’s medium in the presence of 50 ng/ml SCF, 10 ng/ml IL3, and 10 ng/ml IL6 for 24 h. Cells were treated in 75 mM KCl at 37°C for 15–30 min and fixed by addition of ice-cold fixative (1:3 acetic acid:methanol). Metaphase spreads were obtained by dropping fixed cells onto slides. Slides were air-dried overnight and stained with DAPI.

### Data analysis

Fusion mRNAs were analyzed using TopHat software [16]. Frame analysis of fusion mRNA was performed based on our own developed script (Amerlieff, Tokyo, Japan). Functional annotation of RNA sequence data was performed using DAVID Bioinformatics Resources 6.7 [17]. Heat maps and clustering were generated using MeV4.0. Data are expressed as means ± S.E. The Mann–Whitney U test or t-test was used for statistical analysis; *P* values <0.05 were considered significant (*, *P* < 0.05; and ^†^, *P* < 0.01).

## Results

### Fetal concentration of etoposide following maternal exposure

**Fig 1:**
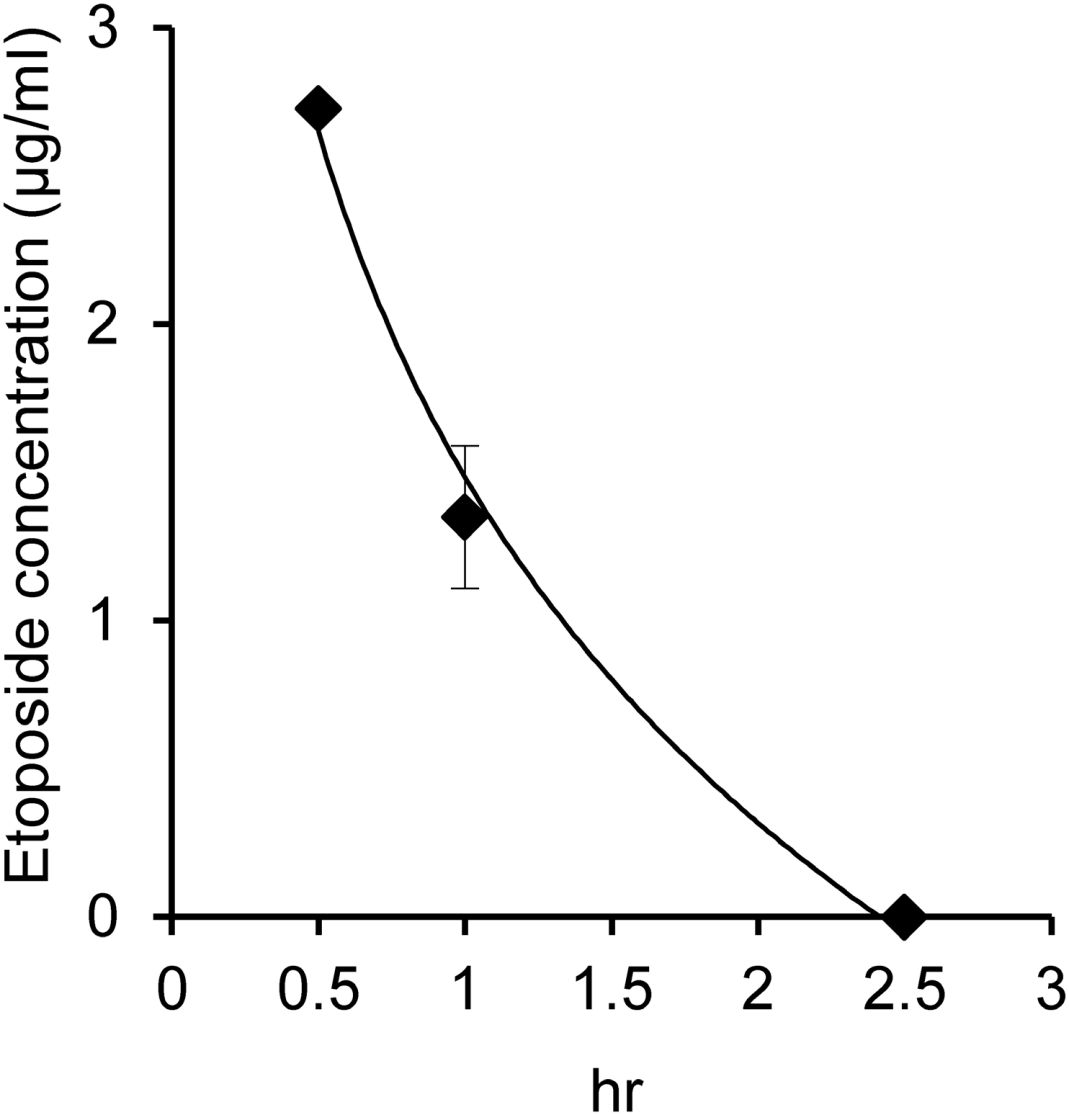
Fetal etoposide concentration after intraperitoneal (IP) etoposide injection. Etoposide (10 mg/kg) was IP injected into E13.5 pregnant mice, and fetal livers were collected at the indicated time points.

Etoposide concentration was measured in fetuses after IP injection of 10 mg/kg etoposide into pregnant female mice on day 13.5. The etoposide concentration in the fetus decreased rapidly, and was undetectable at 2.5 h after the injection (Fig 1); when a dose of 0.5 mg/kg was administered, etoposide was not detectable even immediately after injection (data not shown). The pharmacokinetics data were as follows: area under the blood concentration-time curve (AUC), 266 mg/dl/h; terminal elimination rate constant (Kel), 1.406/h^-1^; elimination half-life (T_1/2_), 0.492 h; volume of distribution (Vd), 0.045 l; clearance (CL), 0.0636 l/h; and clearance total (CL_tot_), 0.0636 l/h. These data suggest that fetuses were exposed to etoposide at a concentration of at least less than 5 μM for 2 h following IP injection of mothers with a dose of 10 mg/kg. However, the effective concentration in fetal cells following maternal injection at a dose of 0.5 mg/kg could not be determined.

### DNA double-strand breaks in the FL-HSC and maternal BM MNC after etoposide injection

**Fig 2:**
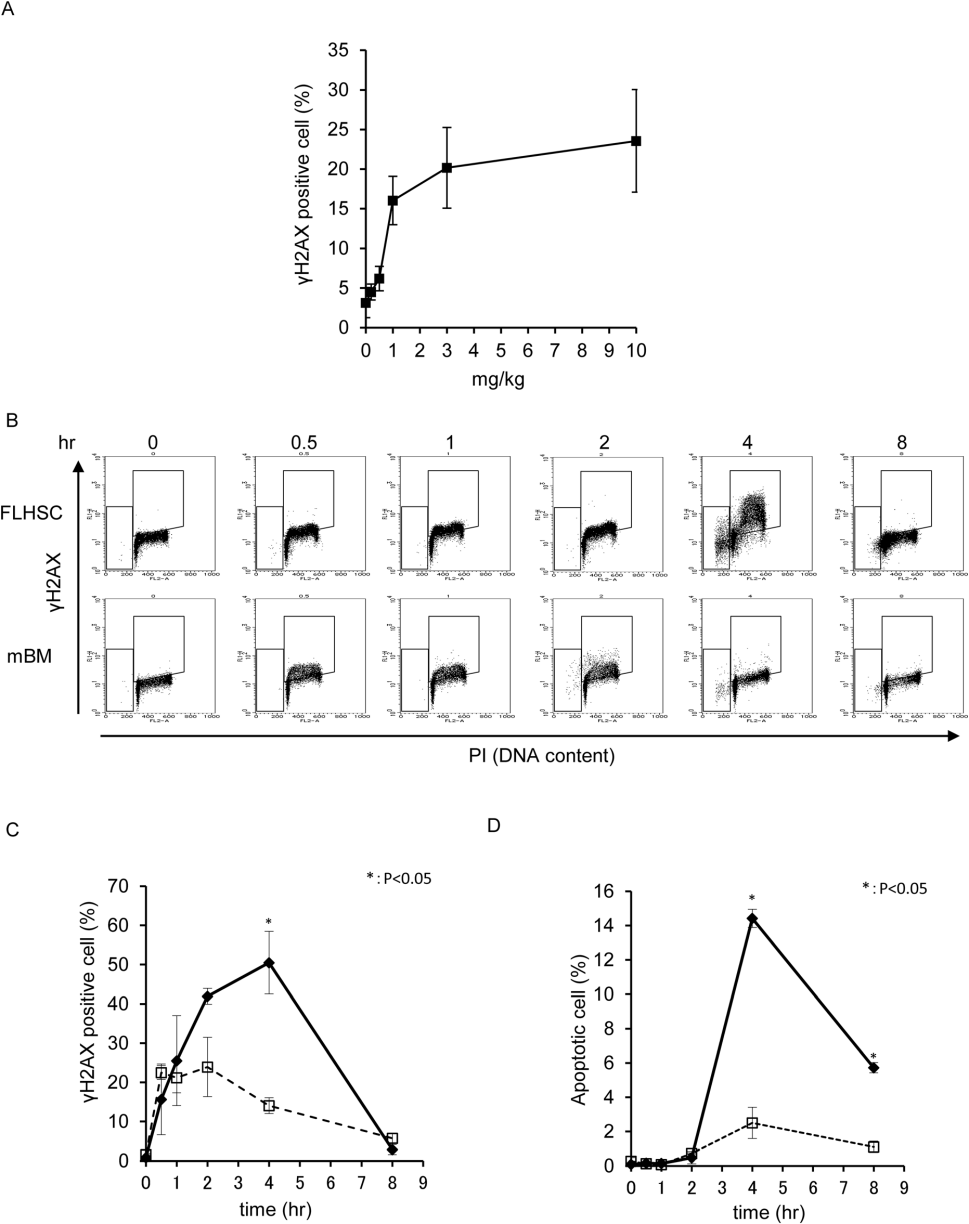
(A) Dose-dependence of γH2AX positivity in fetal liver hematopoietic stem cells (FL-HSCs), analyzed 4 h after IP injection of etoposide at the indicated doses. Etoposide was IP injected into E13.5 pregnant mice. (B) DNA double-strand breaks were detected according to γH2AX positivity, and cell-cycle distribution was monitored by propidium iodide (PI) incorporation. Etoposide (10 mg/kg) was IP injected into E13.5 pregnant mice, and samples were analyzed at the indicated time points. A two-dimensional dot blot is shown. FL-HSC: fetal liver hematopoietic stem cells; mBM: maternal bone marrow mono nuclear cells. (C) The kinetics of γH2AX positivity in the samples shown in B are expressed as a line graph. Bold line indicates FL-HSC, and broken line indicates mBM. (D) Percentage of apoptotic cells in the samples shown in B.

DNA damage was examined in FL-HSCs from pregnant female mice on day 13.5 and maternal BM MNCs in response to IP injection of etoposide into pregnant mice. γH2AX (Serine 139 phosphorylated H2AX) is a molecular marker of DNA damage, including DNA double-strand and single-strand breaks. The percentage of γH2AX-positive cells was measured by flow cytometry. The dose-dependence of DNA damage induction in FL-HSCs was investigated using γH2AX positivity as an indicator. IP injection of 0.2–0.5 mg/kg etoposide into pregnant mice induced minimal DNA damage in the FL-HSC, and γH2AX positivity gradually increased in a dose-dependent manner (Fig 2A). γH2AX induction by low concentrations of etoposide (0.5 mg/kg) was not significantly detectable by flow cytometry; therefore, a relatively high dose of etoposide (10 mg/kg) was used to characterize the *in vivo* effects on the fetus. Kinetic analysis revealed that γH2AX-positive cells were detectable in the FL-HSC and maternal BM MNC immediately after injection of 10 mg/kg etoposide, reaching a peak at 1–2 h in the maternal BM MNC and at 4 h in the FL-HSC. γH2AX-positive cells were more frequent in the FL-HSC than in the maternal BM MNC (Fig 2B and 2C and S1A and S1B Fig). In addition to becoming γH2AX-positive, FL-HSCs exhibited activation of ATM, which plays a central role in the DNA damage checkpoint (S1C Fig). Apoptosis was induced at 2 h after IP injection in maternal BM MNCs and, at a higher rate, in the FL-HSC (Fig 2B and 2D). The percentage of apoptotic cells reached a peak at 4 h after IP injection and decreased over the next 4 h, possibly because of the clearance of dead cells.

### Alteration of the cell cycle following etoposide injection

**Fig 3:**
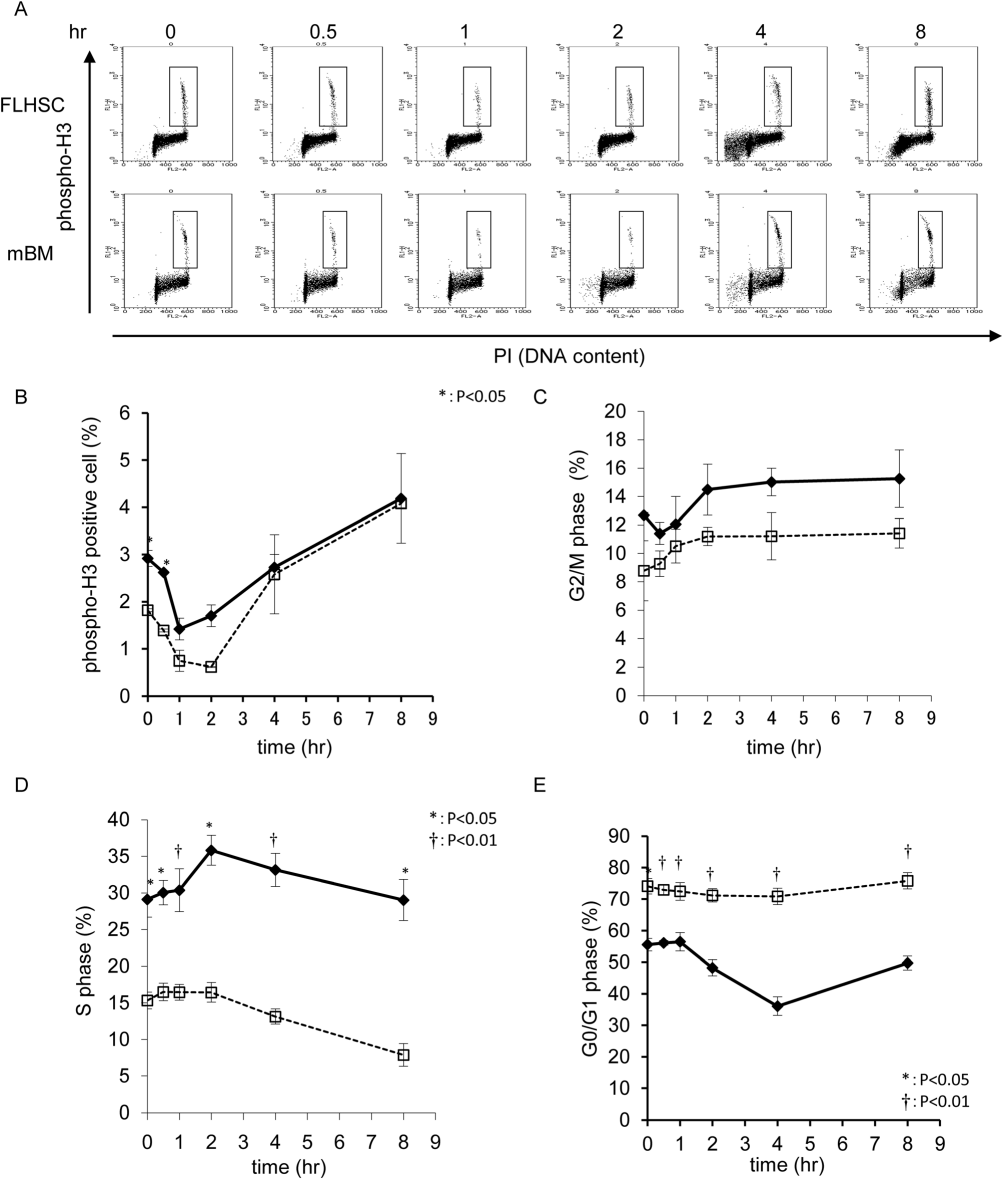
(A) Cell-cycle distribution was monitored by a combination of phospho-histone H3 positivity and PI incorporation. Etoposide (10 mg/kg) was IP injected into E13.5 pregnant mice, and samples were analyzed at the indicated time points. A two-dimensional dot blot is shown. (B) Kinetics of phospho-histone H3 positivity indicating M phase percent, shown as a line graph. Bold line indicates FL-HSC, and broken line indicates mBM. (C) G2/M phase cell percent, (D) S phase cell percent, and (E) G0/G1 phase cell percent.

**Fig 4:**
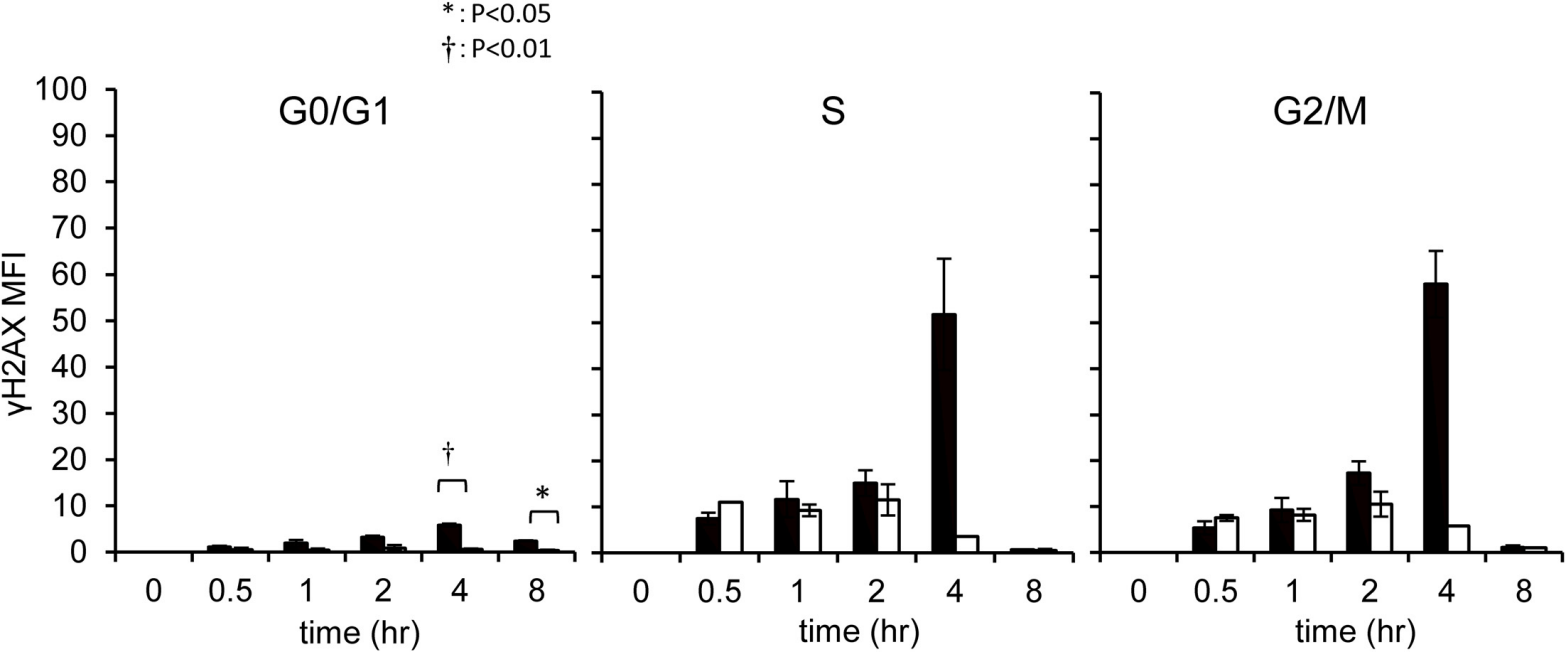
DNA double-strand breaks in FL-HSCs at each phase of the cell cycle. DNA double-strand breaks were monitored by γH2AX positivity. DMSO or etoposide (10 mg/kg) was IP injected into E13.5 mice, and samples were analyzed at the indicated time points. White bar indicates the DMSO injected group. Black bar indicates the etoposide injected group.

The cell cycle is finely regulated by the DNA damage checkpoint. The proportion of S phase cells was higher in FL-HSCs from pregnant mice on day 13.5 than in the maternal BM MNC, indicating that cell-cycle progression occurred at a faster rate in the FL-HSC than in the BM MNC (Fig 3A and 3D). To investigate this further, cell-cycle kinetics were assessed following IP injection of 10 mg/kg etoposide into pregnant mice on day 13.5. TOP2 is essential for DNA decatenation and enables cell-cycle progression from S to M phase; etoposide, a TOP2 inhibitor, blocks cell-cycle progression from G2 to M phase. The proportion of mitotic cells, as indicated by phospho-histone H3 positivity, was transiently reduced at 0.5–4 h after etoposide injection in both FL-HSC and maternal BM MNC (Fig 3A and 3B). In parallel, G2 phase cells gradually accumulated in both tissues (Fig 3C). Concomitant with the transient M phase arrest, the number of S phase cells transiently increased at 3 h after injection, followed by a gradual decrease. This phenomenon was more pronounced in FL-HSCs than in the maternal BM MNC (Fig 3D). After a temporary G1 arrest, the proportion of G1 phase cells was transiently reduced in the FL-HSC. Maternal BM MNCs did not show obvious changes, indicating that the G1 arrest was persistent in this tissue (Fig 3E). To examine the distribution of DNA damage in response to etoposide exposure, γH2AX positivity was monitored in each cell-cycle phase. γH2AX-positive cells were detected between the S and G2/M phases, and were more abundant in FL-HSCs than in the maternal BM MNCs(Fig 4). Although most DNA damage in cells between the S and G2 phases was resolved by 8 h after injection, 2.4% of FL-HSCs in G1 had DNA breaks, whereas only 0.3% of maternal BM cells in G1 phase were γH2AX-positive. This observation suggests that the DNA damage that occurred between the S and G2/M phases was carried over to the next G1 phase.

### *KMT2A* breakage induced by etoposide

**Fig 5:**
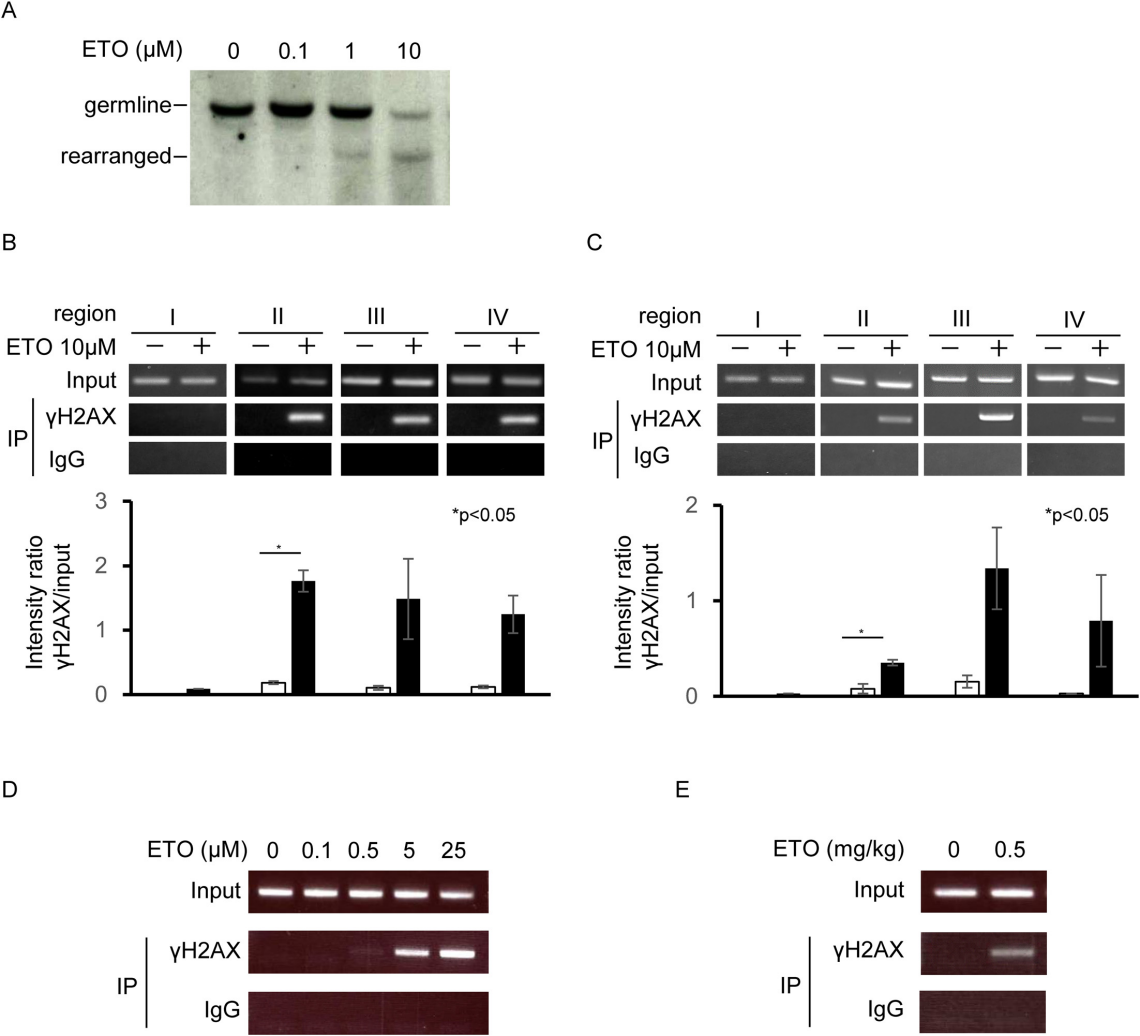
DNA damage at the *KMT2A* gene region after etoposide treatment. (A) Southern blot analysis of human *KMT2A* breakage. Cells were treated with the indicated concentrations of etoposide for 5 h. ETO: etoposide. (B) ChIP analysis of DNA breaks in human *KMT2A*. Cells were treated with 10 μM etoposide for 5 h. (C) ChIP analysis of DNA breaks in mouse *Kmt2a*. (D) Dose-dependent generation of DNA breaks in mouse cells, analyzed by ChIP. Cells were treated with the indicated concentrations of etoposide for 3 h. (F) ChIP analysis of DNA breaks in the fetal mouse *Kmt2a* locus. Pregnant mice were IP injected with 0.5 mg/kg etoposide and analyzed 1 h after injection.

Etoposide causes *KMT2A* rearrangement [18]. Southern blotting is a traditional method for detecting such rearrangements. However, Southern blotting has several limitations, including low sensitivity and a requirement for large amounts of DNA. Therefore, a new method is needed for detecting breaks in *KMT2A*. Because the ChIP assay is a relatively sensitive method for the detection of changes in chromatin, we used this technique to detect DNA breaks in the *KMT2A* locus. γH2AX was used as an indicator of DNA breaks. *In vitro* experiments were first performed to compare the traditional and novel methods; specifically, *KMT2A* rearrangement in BV173 cells was measured by both Southern blotting and ChIP assay. *KMT2A* translocations associated with infant and therapy-related leukemia can be mapped to an 8.3 kb breakpoint cluster region between exons 8 and 11; the probe used to detect *KMT2A* rearrangements by Southern blotting was an 8 kb *Bam*HI fragment spanning nearly that entire region (S2A Fig). Rearranged *KMT2A* was faintly detectable by Southern blotting at 5 h after 1 μM etoposide treatment, whereas it was more prominent after injection of 10 μM etoposide (Fig 5A). ChIP to detect γH2AX on *KMT2A* was performed using four sets of primers: one in exons 9 and two in the vicinity of exon 11. A primer was also designed as a negative control for *KMT2A* breakage at a neighbor gene, *BCL9L*, which is located 400 kb from the *KMT2A* gene (S2A Fig). γH2AX-positive DNA damaged regions were detected using the exon 9 and vicinity of exon 11 primers, which are located within the breakpoint cluster region (Fig 5B), but were not detected with the primers for *BCL9L*. Next, we investigated whether this ChIP assay could detect breaks in *Kmt2a* in mouse Ba/F3 cells *in vitro*. For these experiments, we designed primers against *Kmt2a* (S2B Fig). As same as in human, γH2AX-positive DNA damaged regions were detected in *Kmt2a* gene (Fig 5C). Next, cells were treated with various concentrations of etoposide, and the ChIP assay was performed. DNA breaks were detected at etoposide concentrations of 0.5 μM and higher (Fig 5D). Finally, we investigated DNA breaks *in vivo* in FL-HSCs from pregnant female mice on day 13.5. The ChIP assay detected DNA breaks in *Kmt2a* in FL-HSCs following IP injection of 0.5 mg/kg etoposide into pregnant mice (Fig 5E).

### *In vivo* fetal response to etoposide

**Fig 6:**
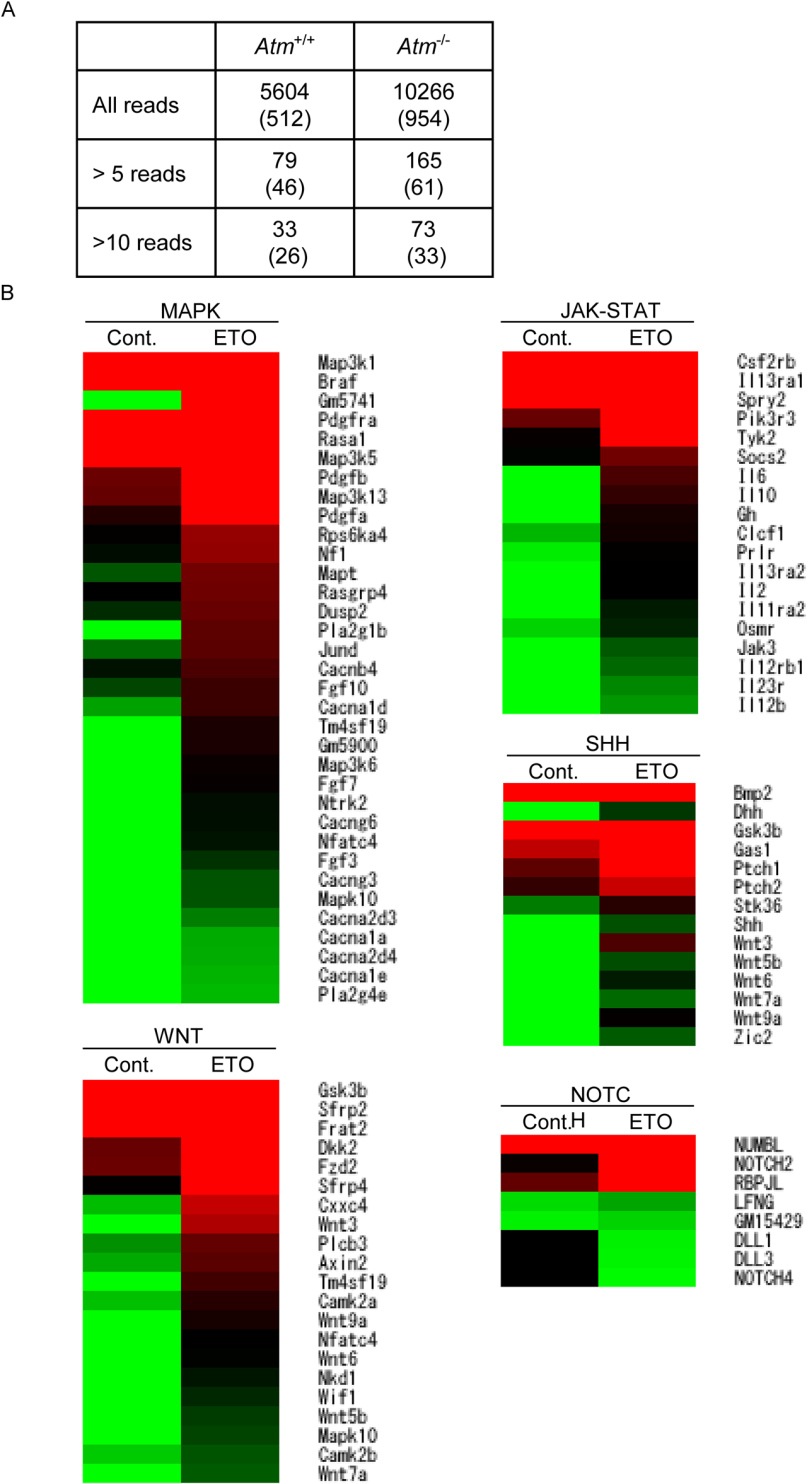
E13.5 pregnant wild-type mice were IP injected with saline or etoposide (0.5 mg/kg) on 3 consecutive days, and FL-HSCs were harvested for expression analysis 24 h after the final injection (E16.5). (A) Number of chimeric mRNAs detected by RNA-seq in *Atm* ^-/-^ mice and wild-type littermates. Numbers in parenthesis indicate in-frame fusion mRNAs. (B) Heat map of selected genes associated with signal transduction, enriched using the DAVID software. Cont. DMSO; treated, ETO; etoposide-treated. Red indicates upregulated and green indicates downregulated expression in color ramp for heatmap

Based on these observations, we postulated that 0.5 mg/kg etoposide is the minimal concentration required to induce DNA damage in the *Kmt2a* region. We then attempted to detect *Kmt2a* breakage after etoposide administration. Mouse *Kmt2a* is located on chromosome 9. Therefore, chromosome painting was performed. After maternal etoposide exposure between days 13.5 and 15.5 of pregnancy, FL-HSCs from day 16.5 of pregnancy were examined, which showed no chromosomal aberrations (0/88). We hypothesized that FISH analysis was not sensitive enough. To precisely evaluate the *in vivo* effect of low-dose maternal etoposide exposure on the fetus, we performed RNA-seq to detect fusion genes and alterations in mRNA expression patterns. After maternal etoposide exposure between days 13.5 and 15.5 of pregnancy, various chimeric mRNAs were detected in FL-HSCs from day 16.5 of pregnancy (S1 Data and Fig 6A). However, chimeric fusion mRNAs involving *Kmt2a* were not detected.

DNA breaks and etoposide treatment itself alter gene expression patterns [19]. Hence, we compared the *in vivo* gene expression profiles of control and etoposide-exposed wild-type FL-HSCs. Several genes were up- or downregulated after etoposide exposure. Pathway analysis of these genes identified 21 upregulated and four downregulated pathways (Fig 6B and S2 Data). Several pathways that accelerate cell proliferation, including the MAPK, WNT, JAK-STAT, SHH and NOTCH pathways, were upregulated after etoposide exposure.

### Cell-cycle dysregulation after DNA damage causes *Kmt2a* rearrangement

**Fig 7:**
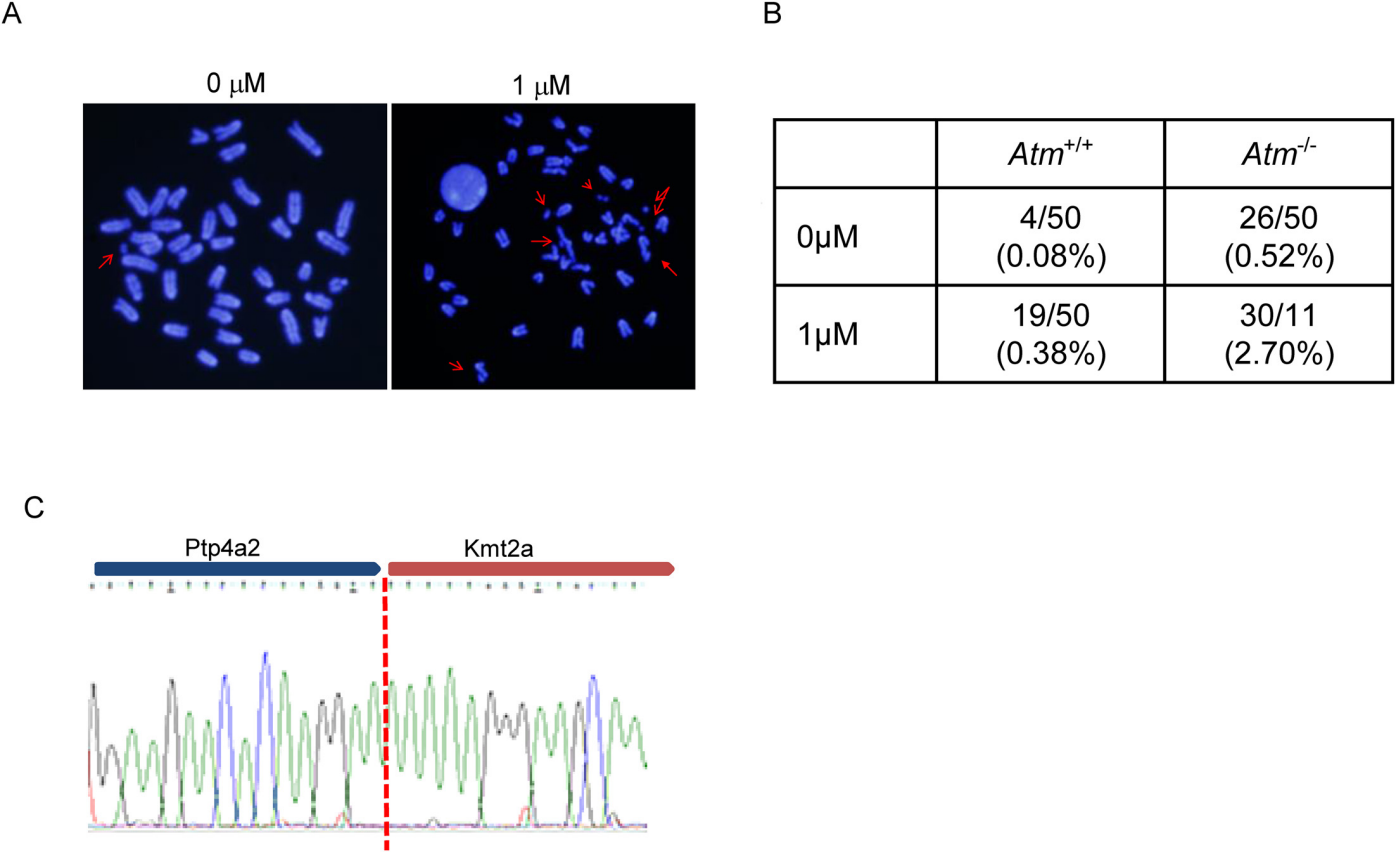
FL-HSCs of *Atm*-knockout mice were pulse-treated with etoposide for 4 h *in vitro*; metaphase spreads were generated 24 h after pulse treatment. (A) Representative metaphase spreads. Arrows indicate chromosomal or chromatid breaks. (B) Number of chromosomal breaks and chromatid breaks, shown as a table. (C) Sequence electropherogram of *Kmt2a*-*Ptp4a2* fusion mRNA.

*Kmt2a* rearrangement after etoposide treatment was not observed in wild-type mice. Our previous *in vitro* study demonstrated that ATM-deficient fibroblasts, which cannot activate the early G2/M checkpoint, induce *KMT2A* rearrangement following low-dose etoposide exposure [14]. Hence, we investigated the effect of etoposide on Atm-deficient FL-HSC, which showed that Atm-deficient FL-HSCs contained elevated levels of chromosome and chromatid breaks following etoposide exposure (Fig 7A and 7B). We also performed RNA-seq, as described in the previous section, on FL-HSCs. Various chimeric fusion mRNAs were detected in wild-type and *Atm*-knockout FL-HSCs in response to etoposide treatment (Fig 6A and S1 Data). Chimeric fusion mRNAs were more abundant in the FL-HSCs of Atm-deficient fetuses than in their wild-type littermates. Intriguingly, a *Kmt2a*-*Ptp4a2* fusion mRNA was detected in Atm-deficient FL-HSCs following etoposide exposure (Fig 7C). However, this chimeric mRNA was not an in-frame gene fusion. Well described fusion mRNAs in infant leukemia such as KMT2A-AFF1 (MLL-AF4), KMT2A-MLLT3 (MLL-AF9) and KMT2A-MLLT1 (MLL-ENL) were not observed in this study. In the absence of DNA damage repair, persistent DNA damage leads to chromosomal rearrangement. Therefore, γH2AX positivity in FL-HSC was analyzed after 24 h of maternal exposure to 10 mg/kg etoposide. However, γH2AX positivity was almost resolved in wild-type and *Atm*-knockout FL-HSCs (S3 Fig).

### Increased leukemia development was not observed after *in utero* etoposide exposure

The effect of low-dose etoposide treatment on pup delivery and leukemia development in the offspring was investigated next. *Atm* ^*+/-*^ mice were crossed with each other, and 0.5 mg/kg etoposide or DMSO was administered for 3 days starting on day 13.5 of pregnancy. The number of delivered pups and the frequency of stillbirths did not differ between the DMSO-treated and etoposide-treated groups. A cross of *Atm* ^+/-^ mice is expected to produce offspring with the *Atm* ^+/+^, *Atm* ^+/-^ and *Atm* ^-/-^ genotypes. The frequency of *Atm* ^+/+^, *Atm* ^+/-^ and *Atm* ^-/-^ pups did not differ between the DMSO-treated and etoposide-treated groups (S4A Fig). Leukemia development in the offspring was expected, especially in *Atm* ^-/-^ mice, as *Atm* ^-/-^ mice develop leukemia/lymphoma spontaneously [20]. In the DMSO-treated group, two of eight *Atm* ^-/-^ mice developed T cell lymphoma. In the etoposide-treated group, three of nine *Atm* ^-/-^ mice developed T cell lymphoma (S4B Fig). The frequency of leukemia development did not differ between the two groups. Leukemia development was not observed in *Atm* ^+/+^ or *Atm* ^+/-^ mice. These observations indicated that maternal etoposide exposure did not induce leukemia development, even in the *Atm* ^-/-^-knockout condition. In the two *Atm* ^-/-^ mice that developed tumors after *in utero* etoposide exposure, the tumors were positive for chromosome 9 translocation, where *Kmt2a* is located. However, these translocations did not involve *Kmt2a* translocation (S5 Fig).

## Discussion

Infant acute leukemia is the human diseases that are initiated during embryogenesis or fetal development. Epidemiological studies suggest that the development of infant leukemia is associated with *in utero* exposure to TOP2 inhibitors, which results in the rearrangement of *KMT2A* [21–23]. *In utero* modifications of both the primary DNA sequence and the epigenetic state are involved in this chromosomal translocation. However, because the access to fetuses is limited, these phenomena cannot be studied in human embryos. Therefore, we performed an *in vivo* analysis using mouse models with or without Atm deficiency to determine whether maternal exposure to a TOP2 inhibitor induces *Kmt2a* rearrangement under certain conditions. In the present study, we also developed a novel method for detecting breaks in *KMT2A* using the ChIP assay.

TOP2A, which is mainly expressed between the S and G2/M phases of the cell cycle, is essential for cell-cycle progression. The TOP2 inhibitor etoposide blocks cell-cycle progression from G2 to M phase and induces cell death. Therefore, it is speculated that cycling cells are more sensitive to etoposide. Previously, we hypothesized that cord blood-derived MNCs would be more sensitive to TOP2 inhibitors than adult peripheral MNCs, and that this hypersensitivity to etoposide is related to the development of infant leukemia. However, sensitivity to etoposide did not differ significantly between cord blood-derived MNCs and peripheral MNCs from children [24]. In the current study, we show that the hematopoietic cells of the fetal liver, the primary organ of fetal hematopoiesis, are more sensitive than BM cells to TOP2 inhibitors. This is likely because most fetal liver hematopoietic cells are actively cycling, whereas cord blood MNCs are in G0/G1 phase. Furthermore, our previous results may have been influenced by the artificial *in vitro* conditions used in that study. The kinetics of cell-cycle changes and γH2AX are different between FL-HSCs and the maternal BM MNCs. γH2AX positivity in G2/M was more prominent in FL-HSCs at 4 h after etoposide injection. This could be attributed to the amount of damaged DNA in G2 phase that is carried over from S phase, or explained by impaired DNA repair during S phase in FL-HSCs.

In the present study, DNA breaks were detected *in vivo* using flow cytometry, and the ChIP assay was used to detect γH2AX at the *KMT2A* locus. Southern blotting and FISH analysis are the standard techniques for monitoring *KMT2A* rearrangements in humans; to date, however, no practical method has been established in a mouse model. Here, we described a ChIP assay that can detect DNA breaks in the *KMT2A*/*Kmt2a* region with high sensitivity. We used this method to reproduce *in vivo KMT2A* rearrangement following etoposide treatment. Previous reports revealed that fetal hematopoietic cell or stem cell exposed to relatively low dose etoposide (0.2 to 0.5μM) induces DNA damage and KMT2A translocation [25] [26]. These results are compatible with our data. Intriguingly Bueno et al. reported continuous exposure of extremely low dose etoposide (0.04μM) followed by 0.2μM initial exposure also induces KMT2A translocation [26]. Although several lines of evidence suggest that *KMT2A* breakage occurs in response to exposure to TOP2 inhibitors *in utero*, reproducing *Kmt2a* translocation *in vivo* has not been attempted to date. Cleaved DNA ends are usually repaired by the non-homologous end joining (NHEJ) or homologous recombination repair (HRR) pathway. Especially in the case of etoposide-induced lesions, DNA breaks are mostly generated between the late S and G2/M phases (Fig 4), when breaks are primarily repaired by HRR. To achieve chromosomal translocations, it seems necessary to introduce additional factors, such as dysregulation of cell-cycle checkpoints or defects in DNA repair pathways, in addition to DNA double-strand breaks. In previous work, we showed that chromosomal translocations involving *KMT2A* occur in ATM-deficient cells following etoposide treatment [14]; in addition, such cells induce chromosomal translocations involving the T cell receptor locus via RAG-dependent VDJ rearrangement during thymocyte maturation [27]. Furthermore, ATM regulates the G2/M transition as well as the HRR pathway. Therefore, loss of cell-cycle regulation or defects in the DNA damage response (due to loss or mutation of DNA damage response factors such as ATM, its target genes, or related molecules) may trigger chromosomal translocations following DNA double-strand breaks. ATM-defective cell lines are hypersensitive to etoposide and this is due to high levels of TOP2A expression [28]. Thus, ATM-dependent regulation of TOP2A might be another factor that influences etoposide sensitivity. In addition, dysfunction of ATM plays an important role in the development of infant leukemia in certain cases [29]. Taken together with these findings, our results suggest that increased sensitivity to TOP2 inhibitors caused by mutations or defects of ATM pathways leads to *KMT2A* rearrangement, ultimately resulting in the development of infant leukemia.

The development of leukemia requires activation of cell proliferation in addition to differentiation blockage. Etoposide exposure stimulates FL-HSC proliferation [12]. In the present study, we analyzed gene expression profiles after etoposide treatment. Pathways involved in cell proliferation, such as MAPK and JAK-STAT, were upregulated. This phenomenon may be explained by the process of regeneration of damaged cells tor tissues. If cells retain DNA damage because of defects in the DNA damage response pathway, such as ATM deficiency, activation of proliferative pathways would enable the cells to proliferate despite the persistence of DNA breakage or rearrangements.

Consistent with this, we detected the *Kmt2a-Ptp4a2* fusion mRNA following *in utero* etoposide exposure only in *Atm*-knockout fetuses by using a highly sensitive method (RNA-seq). The *Kmt2a-Ptp4a2* fusion mRNA has not been described previously. Ptp4a2 is a member of the family of protein tyrosine phosphatases (PTPs), which function as cell signaling molecules, and may play a role in hematopoietic renewal [30]. Although this could be a bystander translocation in this case, it still indicates that changes of chromosomal translocations can be generated.

Our results showed that exposure to a TOP2 inhibitor *per se* is not sufficient for rearrangement of *KMT2A in vivo* in a wild-type animal model. Genetic background, such as mutations in the DNA damage response pathway, may influence the likelihood of *KMT2A* rearrangement. However *KMT2A*-rearranged leukemia development was not observed even in *Atm* ^-/-^ mice. *Kmt2a*-*AF4* knock-in mice develop leukemia after prolonged latency, suggesting that a second hit, which might be induced by a possibly defective DNA damage response, is required for full leukemogenesis [31]. Our data also suggested that *Kmt2a* breakage itself is not sufficient for the full development of infantile leukemia, even if the DNA damage response is defective. Infant leukemia has one of the lowest frequencies of somatic mutations of any sequenced cancer [32]. Activating mutation of genes associated with cellular proliferation such as RAS mutations has been identified as an one of these mutations. A defective DNA damage response might be involved in *KMT2A* rearrangement. However, activation of cellular proliferation by mutation of other genes associated with cellular proliferation as well as *KMT2A* rearrangement might be necessary for the full development of leukemia. Taken together, these findings suggest that the identification of other factors in addition to *KMT2A* rearrangement is necessary to improve our understanding of the mechanisms underlying the development of infant leukemia.

## Supporting Information

S1 Data Fusion genes detected by RNA seq. (XLSX)

S2 Data Pathway analysis results. (XLSX)

S1 Fig Supporting data for Fig 1. (PDF)

S2 Fig Supporting data for Fig 1. (PDF)

S3 Fig Western blot analysis of γH2AX positivity 24 hr after etoposide treatment. (PDF)

S4 Fig in vivo effect of etoposide for mice delivery and pups survival. (PDF)

S5 Fig FISH analysis of tumor. (PDF)
