# Supplementary material for: Dysregulation of the DNA Damage Response and KMT2A Rearrangement in Fetal Liver Hematopoietic Cells
Source: PLoS One. 2015 Dec 11;10(12):e0144540. doi: 10.1371/journal.pone.0144540 (PMC4686171; doi:10.1371/journal.pone.0144540)
Supplement: S1 Fig — (PDF) [file pone.0144540.s003.pdf]

Fig S1

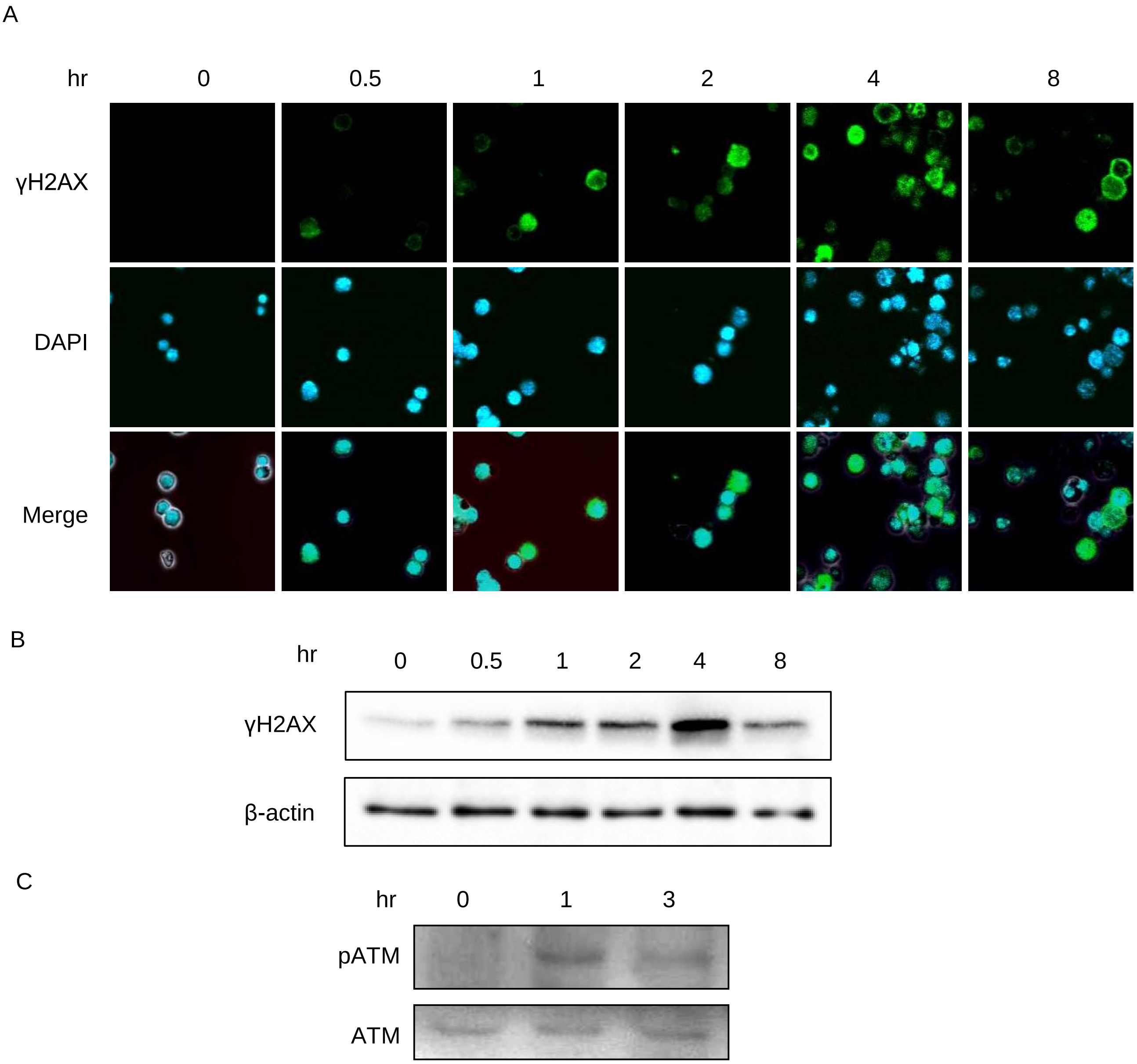

Supplementary figure 1
(A) Immunofluorescence study of γH2AX positivity. Green fluorescence indicates positivity for γH2AX. Blue fluorescence indicates DAPI staining for nucleus. Experimental conditions were the same as those in Figure 2B and C. Etoposide (10 mg/kg) was IP injected into E13.5 pregnant mice, and FL-HSCs were analyzed at the indicated time points (hr). (B) Western blot analysis of γH2AX positivity. Experimental conditions were the same as those in Figure 2B and C. Etoposide (10 mg/kg) was IP injected into E13.5 pregnant mice, and FL-HSCs were analyzed at the indicated time points (hr). (C) Western blot analysis of ATM activation. Etoposide (10 mg/kg) was IP injected into pregnant female mice on day 13.5, and FL-HSCs were analyzed at the indicated time points (hr).
