# Supplementary material for: Dysregulation of the DNA Damage Response and KMT2A Rearrangement in Fetal Liver Hematopoietic Cells
Source: PLoS One. 2015 Dec 11;10(12):e0144540. doi: 10.1371/journal.pone.0144540 (PMC4686171; doi:10.1371/journal.pone.0144540)
Supplement: S2 Fig — (PDF) [file pone.0144540.s004.pdf]

Fig S2

A

NCBI Homo sapiens Annotation Release 107, 2015-03-13

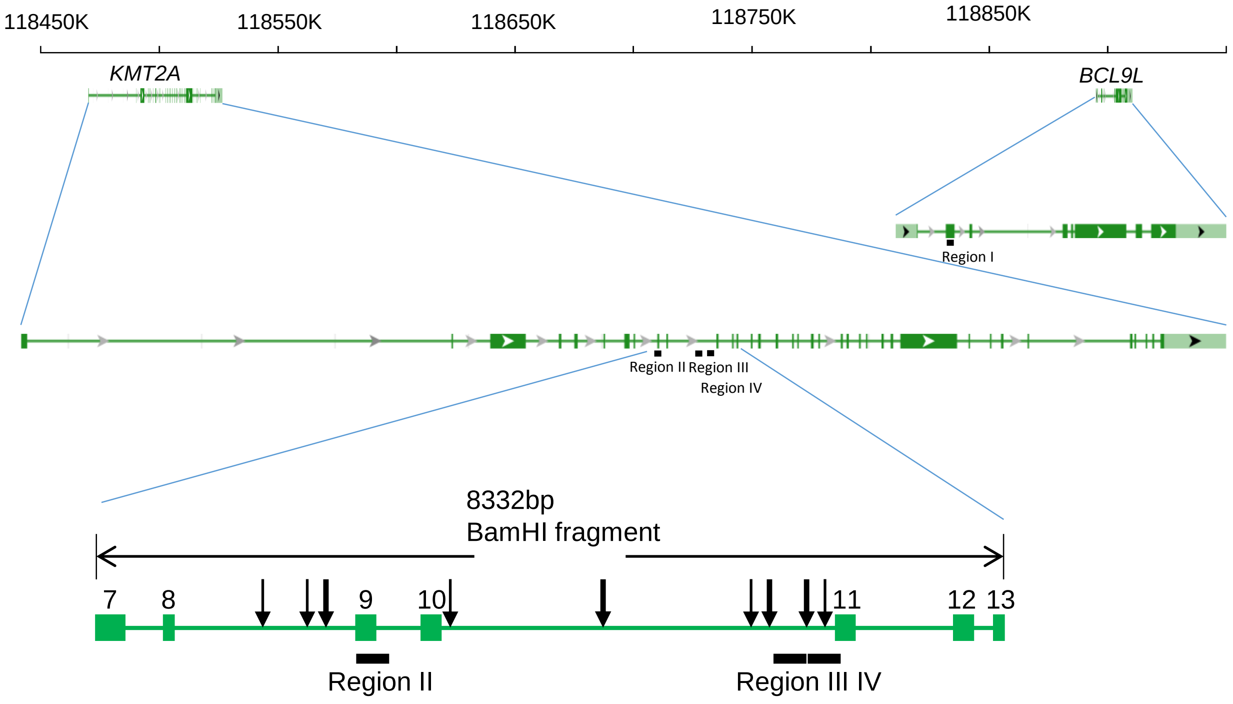

B

NCBI Mus musculus Annotation Release 105, 2015-02-10

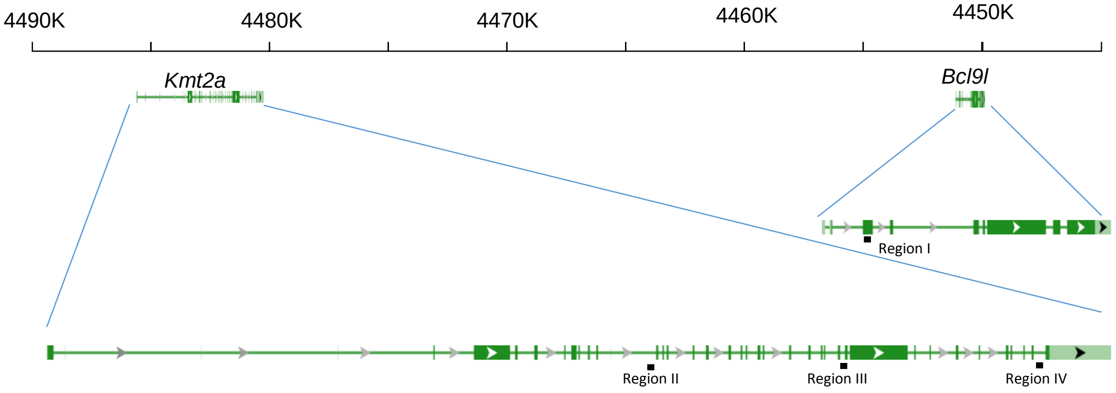

Supplementary figure 2

A andB, Genome map and location of the *KMT2A* or *Kmt2a*, *BCL9L*, or *Bcl9l* genes based on GRCh38 and GRCm38. Green boxes indicate exons. A light green box indicates the untranslated region of the exon. Primer regions are shown in black boxes. Arrows indicate previously reported breakpoint regions.
