# Supplementary material for: Dysregulation of the DNA Damage Response and KMT2A Rearrangement in Fetal Liver Hematopoietic Cells
Source: PLoS One. 2015 Dec 11;10(12):e0144540. doi: 10.1371/journal.pone.0144540 (PMC4686171; doi:10.1371/journal.pone.0144540)
Supplement: S3 Fig — (PDF) [file pone.0144540.s005.pdf]

Fig S3

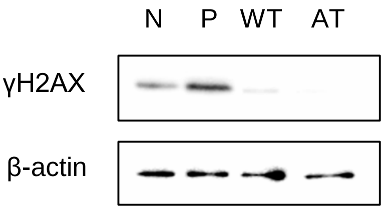

Supplementary figure 3
Western blot analysis of γH2AX positivity. Etoposide (10 mg/kg) was IP injected into pregnant female mice on day 13.5, and samples were collected 24 h after injection. N: negative control using BV173 cells; P: positive control using BV173 cells after 10 μM etoposide treatment for 3 h.
