# Supplementary material for: Dysregulation of the DNA Damage Response and KMT2A Rearrangement in Fetal Liver Hematopoietic Cells
Source: PLoS One. 2015 Dec 11;10(12):e0144540. doi: 10.1371/journal.pone.0144540 (PMC4686171; doi:10.1371/journal.pone.0144540)
Supplement: S4 Fig — (PDF) [file pone.0144540.s006.pdf]

Fig S4

A

	DMSO treated mouse					Etoposide treated mouse				
	Number of pups					Number of pups				
	total	$Atm^{+/+}$	$Atm^{+/-}$	$Atm^{-/-}$	stillbirth	total	$Atm^{+/+}$	$Atm^{+/-}$	$Atm^{-/-}$	stillbirth
exp.1	8	2	3	3	0	8	0	5	3	0
exp.2	9	3	2	2	2	8	2	5	0	1
exp.3	10	4	3	2	1	7	3	3	0	1
exp.4	6	1	5	0	0	5	1	1	2	1
exp.5	6	0	0	0	6	6	2	3	0	1
exp.6	6	0	4	1	1	7	3	3	1	0
exp.7	9	0	0	0	9	5	2	2	1	0
exp.8						10	2	3	2	3
Total	54	10	17	8	19	56	15	25	9	7
Average	7.71	1.43	2.43	1.14	2.71	7	1.88	3.13	1.13	0.88
percent		18.52	31.48	14.81	35.19		26.79	44.64	16.07	12.5

B

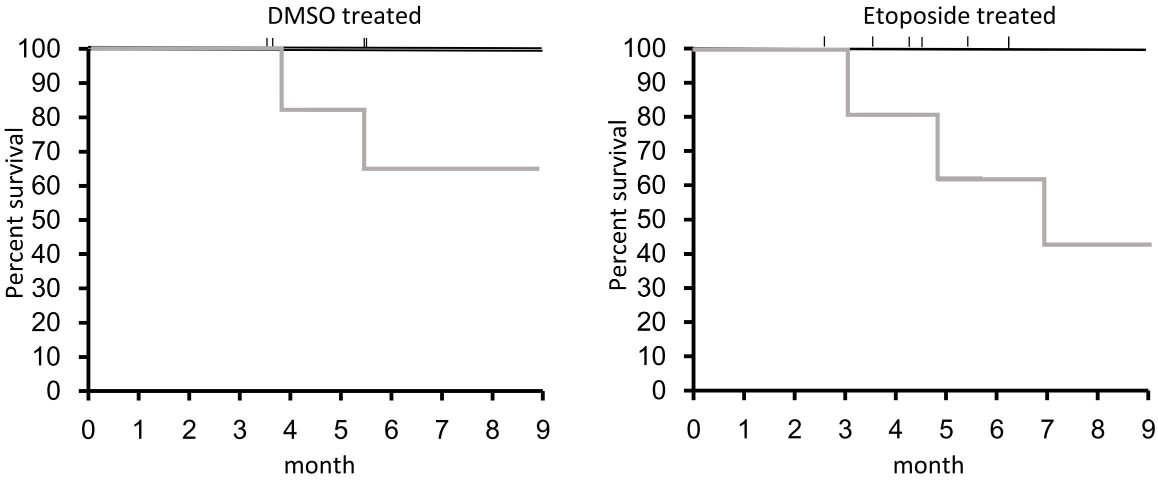

Supplementary figure 4

(A) Number of pups derived by crossing *Atm*$^{+/-}$ mice after DMSO or etoposide treatment. Etoposide (0.5 mg/kg) or DMSO was administered for 3 days starting on day 13.5 of pregnancy. (B) Survival of pups derived by crossing *Atm*$^{+/-}$ mice after DMSO or etoposide treatment. Etoposide (0.5 mg/kg) or DMSO was administered for 3 days starting on day 13.5 of pregnancy. Grey line indicates *Atm*$^{-/-}$ mice. Black line indicates *Atm* $^{+/+}$ mice. Black dotted line indicates *Atm* $^{+/-}$ mice (overlapped with black line).
