# Supplementary material for: Dysregulation of the DNA Damage Response and KMT2A Rearrangement in Fetal Liver Hematopoietic Cells
Source: PLoS One. 2015 Dec 11;10(12):e0144540. doi: 10.1371/journal.pone.0144540 (PMC4686171; doi:10.1371/journal.pone.0144540)
Supplement: S5 Fig — (PDF) [file pone.0144540.s007.pdf]

Fig S5

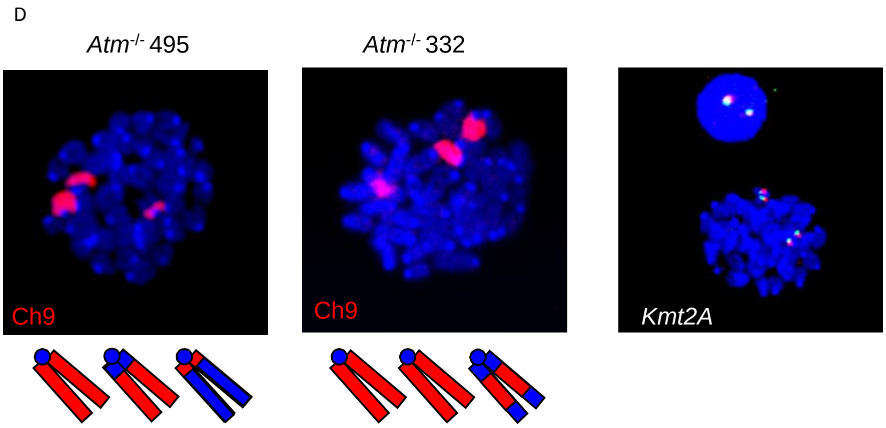

Supplementary figure 5
Chromosomal painting and FISH analysis of leukemic cell developed in *Atm*$^{-/-}$ mice. Chromosome 9 was stained with a red colored probe. Two color FISH probe was designed biterminal of the *Kmt2A* gene.
